# Oil spill detection on X-band marine radar images based on sea clutter fitting model

**DOI:** 10.1016/j.heliyon.2023.e20893

**Published:** 2023-10-11

**Authors:** Peng Liu, Bingxin Liu, Ying Li, Peng Chen, Jin Xu

**Affiliations:** a Navigation College, Dalian Maritime University, Dalian, 116026, China; b Environmental Information Institute, Dalian Maritime University, Dalian, 116026, China; c Maritime College, Guangdong Ocean University, Zhanjiang, 524088, China

**Keywords:** X-band marine radar, Radar images, Oil spill detection, Sea clutter fitting

## Abstract

Oil spills could cause great harm to the natural environment. The ability to identify them accurately is critical for prompt response and treatment. We proposed a sea clutter fitting model of marine radar images for oil spill detection. The model is derived from the geometric structure of the marine radar, the expression of marine radar received power, and the rough surface scattering model of the sea surface. In the denoised marine radar image, the sea clutter fitting model is used to detect coarse oil spills. Then the fine measurement is carried out by mean filter, the Otsu method, and noise reduction. The proposed oil spill detection method was used on radar images sampled after an oil spill accident happened in a coastal region in Dalian, China, on July 21, 2010. The proposed method can detect oil spills without human intervention, and the extracted oil spills are accurate and consistent with visual interpretation.

## 1 Introduction

Oil spills cause immediate and long-term environmental and ecological damage. In oil spill accidents, crude oil at and beneath the water's surface can quickly spread and reach the coastal region under particular weather and oceanographic conditions [[1], [2], [3], [4], [5]]. Therefore, quick and accurate oil spill detection is a critical step for oil spill cleaning. Marine radars are normally installed in vessels for navigation [6]. Hence, the marine radar images used in this study are convenient and expedient to obtain. In marine radar images, the intensity of the backscattered signal in an oil spill area is weaker than that in neighboring waters, a phenomenon that can be exploited to detect oil spills [7]. Although several commercial systems have been developed, such as the oil spill detection (OSD) system of Miros (Norwegian company) [8,9], the SeaDarQ radar system of Nortek Netherlands [10] and sigma S6 OSD system of Rutter (Canada company) [11], oil spill extraction methods are seldom publicized due to commercial competition.

In the 1980s, some scholarly studies on how to use marine radar to detect oil spills were published [12,13], but the oil spill segmentation method was not introduced. Adaptive thresholding methods [14,15] were used to visualize the oil spills on individual sampled radar images. In their study, a manually set threshold had to be used to select an available area for oil spill segmentation. Replacing the available area, texture analysis and machine learning methods were used to carry out coarse measurements of oil spills [[16], [17], [18]] in navigation radar images. However, thresholds for texture analysis and training data for machine learning methods were set manually. An active contour model [19,20] was also introduced to extract oil spills in marine radar images. In a manually selected local area containing oil spills, an active contour model can identify the oil spill precisely. These methods required selecting the appropriate area manually for oil spill extraction. Artificial intelligence methods have been well developed, some machine learning and deep learning methods were used for oil spill detection and drift prediction. Deep convolutional neural networks were used to detect oil spill based on Sentinel-1 SAR images [21]. An adversarial ConvLSTM network framework was used for correcting forecasted wind fields and oil spill drift prediction based on Sentinel-2 satellite images [22]. A Soft Attention Segmentation Model was introduced to carry out oil spill detection based on marine radar images [23]. Artificial intelligence methods require large amounts of training data. However, it is difficult to provide training data of marine radar images containing oil spills in various sea conditions.

Zhu et al. proposed a method to detect oil spills without region selection [24]. In their study, the attenuation of radar signal intensity in each marine radar image was estimated by multiple continuously collected radar images, and a threshold was manually set to extract the oil spills. In this method, it ignores the influence of the ship's location and direction. Another disadvantage is that the calculation of the signal intensity attenuation requires an extended period of time and a large number of images. Replacing multiple radar images, an average of the gray value in the radar image at the same distance was used as the attenuated signal intensity [15]. However, this method ignored the effect of wave propagation direction on radar echo.

In this paper, we propose a sea clutter fitting model of navigation radar image, which is calculated in each incident direction. Based on this model, an oil spill detection method is developed. Section 2 describes the derivation process of the sea clutter fitting model. Section 3 explores an oil spill measurement method. First, the navigation radar image is preprocessed by coordinate transformation and noise reduction. Then, the difference between an estimated radar image by a sea clutter fitting model and a preprocessed radar image is obtained. Finally, oil spills are detected from the difference by Otsu method [25,26] and noise reduction. In Section 4, oil spill detection is carried out using the proposed method on several radar images, and the performance of the proposed method is discussed with a polynomial fitting method and an adaptive thresholding method. Finally, Section 5 presents the conclusions.

## 2 Sea clutter fitting model

**Fig. 1:**
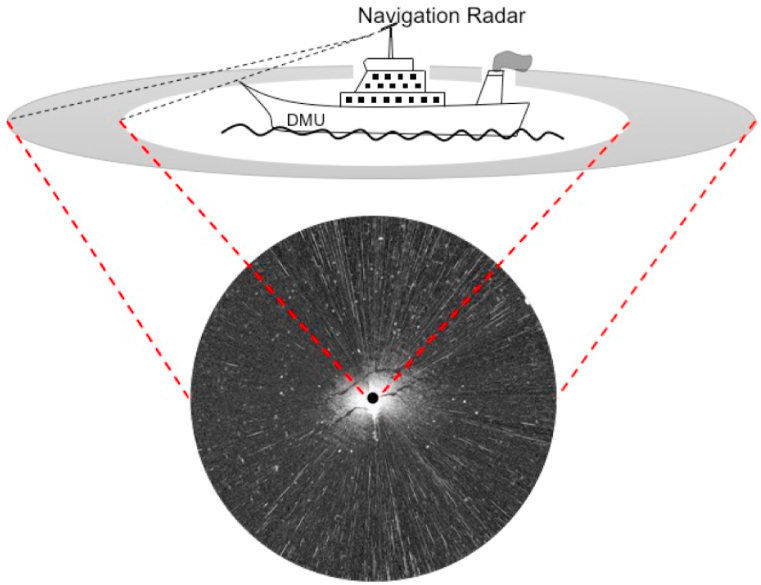
The structure of navigation radar.

**Fig. 2:**
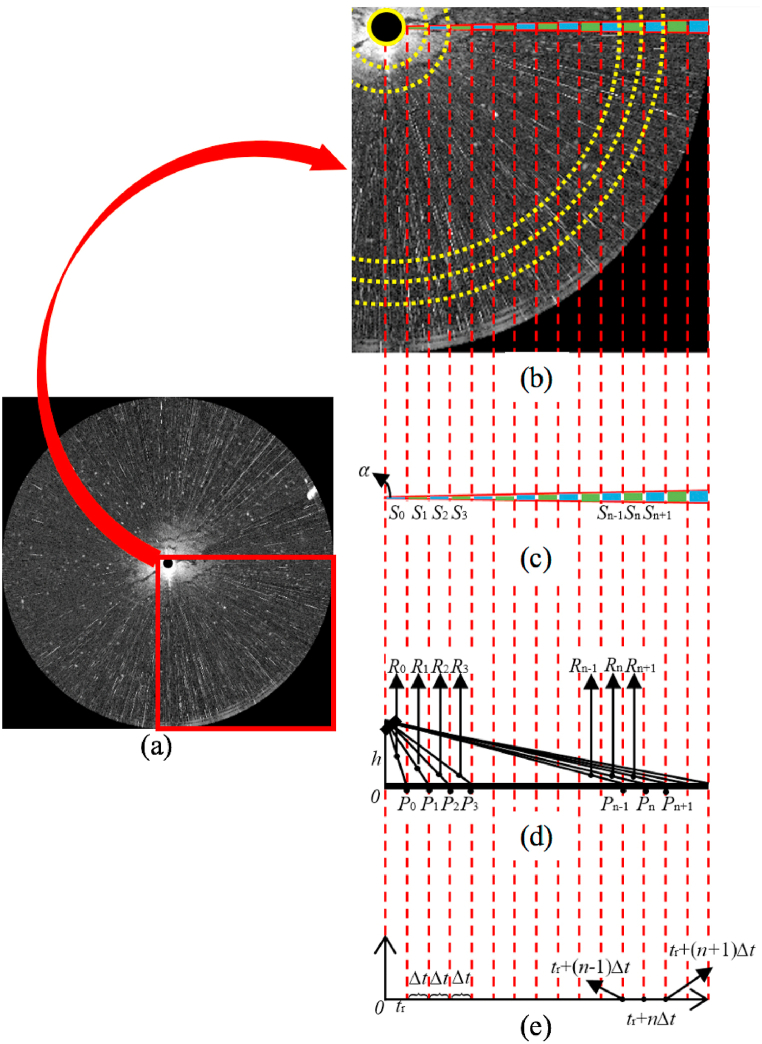
The relationship between the received navigation radar signal in time series and images; (a) marine radar image; (b) part of the marine radar image; (c) sea surface area represented by each pixel in the incident direction; (d) schematic diagram of the signal transmission distance; (e) schematic diagram of received echo in time series.

The structure of navigation radar is shown in Fig. 1. According to the structure of navigation radar, the relationship between the received navigation radar signal in time series and images is shown in Fig. 2. The lower right part of the received radar image (Fig. 2a) is enlarged in Fig. 2b. The value of each pixel on the radar image represents the amount of backscatter energy. In the direction of detection, the sea surface reflection area represented by each pixel in the radar image is related to distance. An example in the horizontal direction of Fig. 2b is shown in Fig. 2c. The time delay of a radar echo is determined by the transmission distance. The schematic diagram of the signal transmission distance and the received echo in time series are shown in Fig. 2d and e, respectively.

As shown in Fig. 2e, $t_{r}$ is the time delay of the echo from the border of the blind area, and the echo shown in the radar image is the closest effective pixel. Corresponding to $t_{r}$, the transmission distance $R_{0}$ (shown in Fig. 2d) can be expressed as follows:

(1) $$R_{0}=\frac{1}{2}Ct_{r}\text{,}$$

where C is the transmission speed of microwave.

The time delay corresponding to each pixel is set as Δt, then the time delay corresponding to the n th pixel is

(2) $$t_{r}\left(n\right)=t_{r}+n\Delta t\text{,}$$

and the transmission distance during the time delay Δt is

(3) $$L=\frac{1}{2}C\Delta t\text{.}$$

Based on Equation (1), Equation (2) and Equation (3), the corresponding transmission distance is

(4) $$R_{n}=\frac{1}{2}Ct_{r}\left(n\right)=\frac{1}{2}C\left(t_{r}+n\Delta t\right)=R_{0}+nL\text{,}$$

As shown in Fig. 2c, suppose the horizontal antenna beam width is α, the reflected sea surface area ($S_{n}$) corresponding to the n th pixel is:

(5) $$S_{n}=\frac{\alpha }{2\pi }\left(\pi {\bar{OP_{n}}}^{2}-\pi {\bar{OP_{n-1}}}^{2}\right)$$

where $\bar{OP_{n}}$ is the distance from ship to the reflected point, which is shown in Fig. 2d. Based on the structure in Fig. 2d, the distance $\bar{OP_{n}}$ can be expressed as:

(6) $$\bar{OP_{n}}=\sqrt{{R_{n}}^{2}-h^{2}}$$

where h is the height of the radar antenna.

Bring Equation (4) and Equation (6) into Equation (5), new expression of Equation (5) can be obtained as

(7) $$S_{n}=\frac{\alpha L}{2}\left(2R_{n}-L\right)=K_{s}^{0}+K_{s}^{1}R_{n}$$

where

(8) $$K_{s}^{0}=-\frac{\alpha L^{2}}{2}$$

(9) $$K_{s}^{1}=\alpha L$$

According to Equation (8) and Equation (9), the values of $K_{s}^{0}$ and $K_{s}^{1}$ are determined for each marine radar.

The power of radar receiver ($P_{r}$) can be expressed as [27]：

(10) $$P_{r}=\frac{P_{t}G_{t}}{4\pi R^{2}}\sigma \frac{A_{r}}{4\pi R^{2}}=K_{P}\frac{\sigma }{R^{4}}\text{,}$$

where.

$P_{t}$ is the power of the radar transmitter;

$G_{t}$ is the power gain of a transmitting antenna; R is the range; σ is the radar cross section (RCS); $A_{r}$ is the effective aperture area; $K_{P}$ is the coefficient calculated as following:

(11) $$K_{P}=\frac{A_{r}P_{t}G_{t}}{{\left(4\pi \right)}^{2}}\;\text{.}$$

For an installed navigation radar system, the parameters of antenna are set and known for users. Therefore, $P_{t}$, $G_{t}$ and $A_{r}$ are known, and $K_{P}$ which calculated by $P_{t}$, $G_{t}$ and $A_{r}$ in Equation (11) is also known. The received power of navigation radar $P_{r}$ is inverse proportional to $R^{4}$ and proportional to RCS (σ). The transmission distance for the n th pixel in the incident direction ($R_{n}$) is shown in Equation (3). The RCS of the area represented by the n th pixel in the incident direction ($\sigma_{n}$) can be expressed as:

(12) $$\sigma_{n}=\sigma_{n}^{0}S_{n}$$

where $\sigma_{n}^{0}$ is the normalized RCS.

**Fig. 3:**
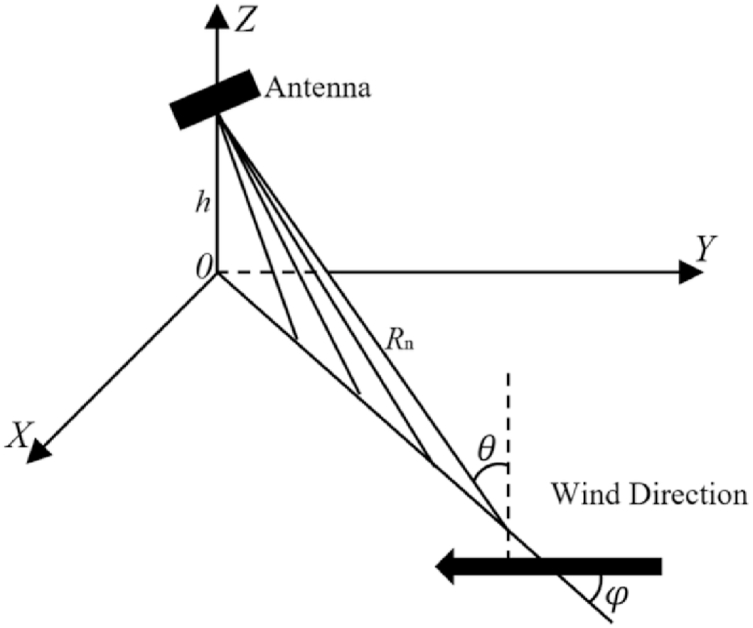
Schematic diagram of the rough surface scattering model.

The sea clutter RCS model is affected by grazing angle, wind direction, polarization and sea state, and the schematic diagram is shown in Fig. 3.

For a low grazing angle, there are several empirical normalized sea clutter RCS models, such as the model proposed by Horst et al. [28], by Reilly and Dockery [29]. For a medium and high grazing angle, Ulaby et al. proposed an empirical normalized sea clutter RCS model above 30 ° [30].

Because the sea surface covered by the radar image is limited, the wind and sea state are considered steady. Therefore, for each polarization, the empirical models of normalized sea clutter RCS are determined by the grazing angle. As shown in Fig. 3, the grazing angle (θ) can be expressed as

(13) $$\tan\;\theta =\frac{h}{R_{n}}$$

Based on Equation (13), the expressions of empirical models can be expressed as functions of $R_{n}$. In this research, M th order Taylor polynomial of the empirical models by $R_{n}$ are used, then the RCS can be expressed as

(14) $$\sigma_{n}^{0}\left(R_{n}\right)=\sum_{\eta =0}^{M}C_{\eta }{R_{n}}^{\eta }$$

where $C_{\eta }$ is the coefficient.

Bring Equation (7), Equation (12) and Equation (14) into Equation (10), the power of radar receiver on the nth pixel in the look direction can be expressed as

(15) $$P_{r}\left(n\right)=K_{P}\frac{\sigma_{n}^{0}\left(R_{n}\right)S_{n}}{{R_{n}}^{4}}=\frac{\sum_{\eta =0}^{M+1}D_{\eta }{R_{n}}^{\eta }}{{R_{n}}^{4}}=\sum_{\eta =-4}^{M-3}D_{\eta }{R_{n}}^{\eta }\text{,}$$

where $D_{\eta }$ is the coefficient.

## 3 Oil spill detection model

**Fig. 4:**
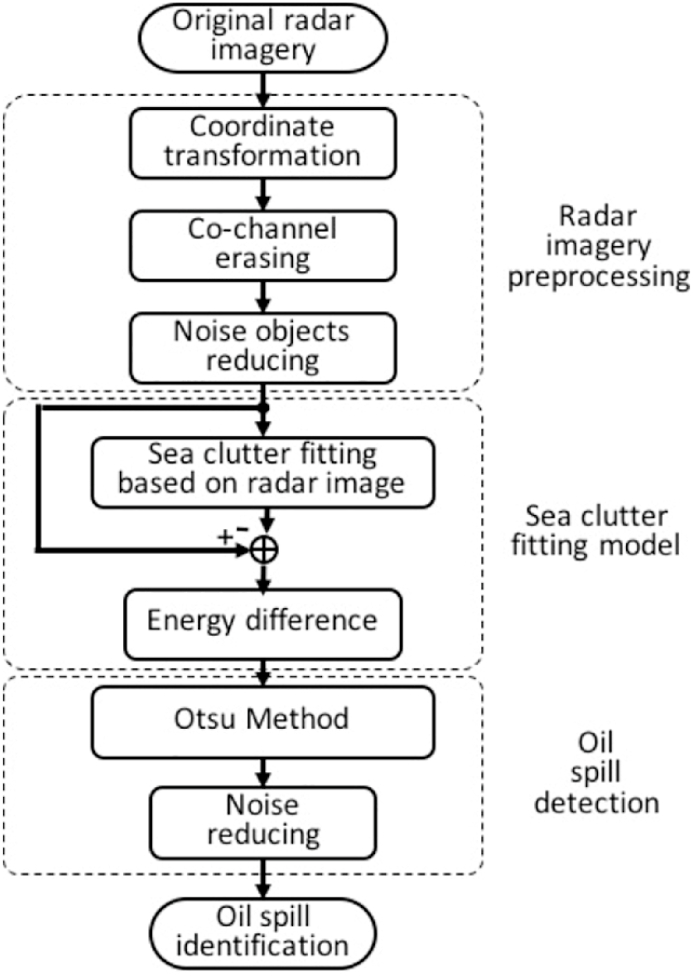
Flow chart of oil spill detection method using radar sea clutter fitting model.

Based on the sea clutter fitting model, data processing for oil spill detection is shown in Fig. 4. First, the sampled marine radar image is preprocessed, specifically in terms of coordinate transformation, co-channel interference erasing, and bright speckle reduction. Then, according to the sea clutter fitting model of the radar image, the difference between the estimated radar image and the preprocessed radar image is obtained. Finally, based on the Otsu method and noise reducing, oil spills can be identified.

### 3.1 Radar image preprocessing

**Fig. 5:**
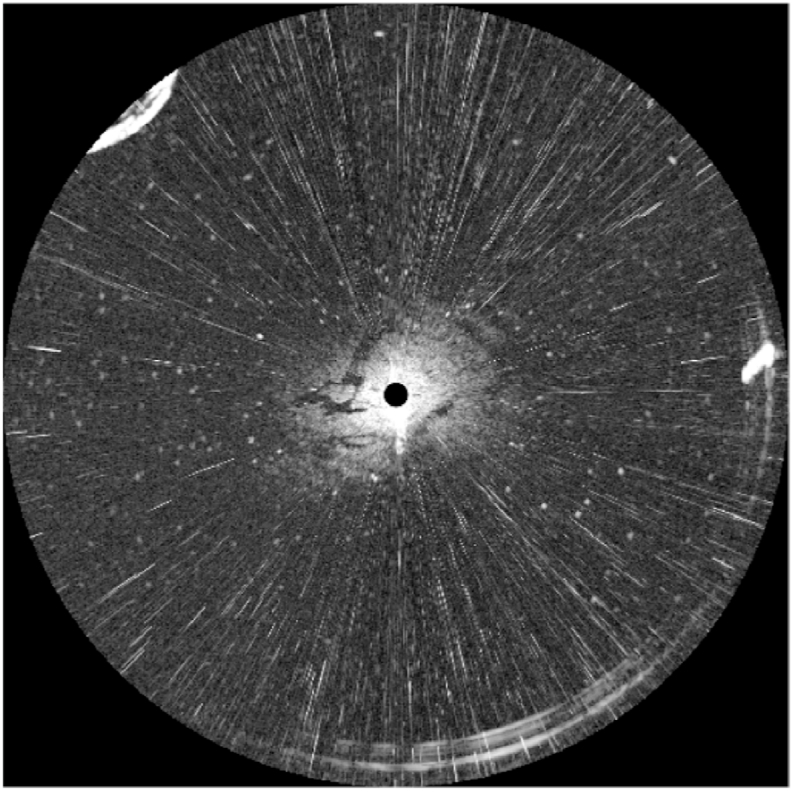
Navigation radar image.

**Fig. 6:**
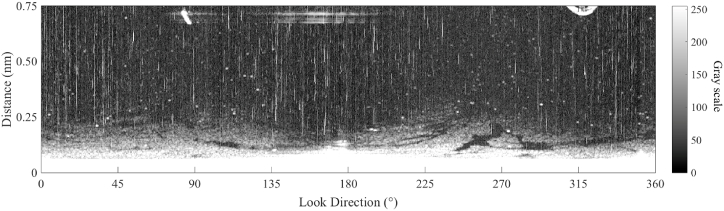
Transformed Navigation radar image.

The radar image of Fig. 5 was sampled at the seashore near Dalian Bay. The radar detection radius in Fig. 5 is 0.75 nautical miles. Fig. 5 is a typical radar image for oil spill detection, which includes oil spills, as well as land, co-channel interference, and bright speckles. For a more straightforward analysis, the radar image of Fig. 5 is transformed into Fig. 6. In Fig. 6, the horizontal axis is the transmitting angle of the marine radar in horizontal, the vertical axis indicates the distance from the radar antenna. The gray value of Fig. 6 presents the energy of the reflected signal. The image size of Fig. 6 is 512 × 2048.

In Fig. 6, bright bands are generated by co-channel interference, which affects the quality of the radar image. To erase the co-channel interference, an index is introduced. The index is calculated by two convolutional filters [14], which is expressed as following:

(16) $$C\left(i,j\right)=\frac{\sum_{k=-\left(N-1\right)/2}^{\left(N-1\right)/2}I\left(i,j+k\right)V_{c}\left(k\right)}{\sum_{l=-\left(N-1\right)/2}^{\left(N-1\right)/2}I\left(i+l,j\right)V_{r}\left(l\right)}$$

where C(i,j) is the index value; $V_{c}$ is the N by 1 convolutional filter; $V_{r}$ is the 1 by N convolutional filter; N is the length of convolutional filter, which is set as an odd number.

**Fig. 7:**
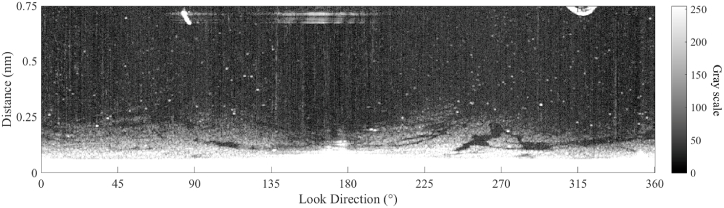
Radar image with co-channel interference removed.

According to Equation (16), the index value of co-channel interference is larger than others. Then, the co-channel interference can be identified and extracted by the Otsu method. To erase the identified co-channel interference, a 1 by N matrix of a mean filter is used [31]. The image erasing co-channel interference of Fig. 6 is shown in Fig. 7. The co-channel interference of Fig. 6 is identified by the index value calculated by two convolutional filters [1, 1, 1, 1, 1, 1, 1] and [1; 1; 1; 1; 1; 1; 1], and suppressed by the mean filter [1/6, 1/6, 1/6, 0, 1/6, 1/6, 1/6]. Based on the proposed index value, Otsu method, and mean filter, only the value of the pixel at the loci of co-channel interference is modified.

**Fig. 8:**
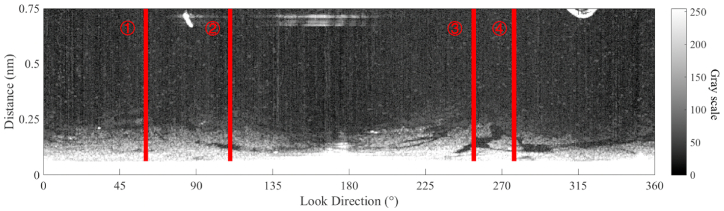
Radar image with co-channel interference and bright speckles reduced.

After suppressing the co-channel interference, Fig. 7 still contains bright speckles, which disturb future oil spill detection. Therefore, the Fields-of-Experts (FoE) model [32] is used to reduce the bright speckles. In the FoE model, bright speckles are considered prior information. To obtain the information of bright speckles, first, Otsu method is introduced to get the binarization of the radar image, and then connected component analysis (less than 200 pixels) is used to extract the speckles. Bright speckles are suppressed by using 8 filters of 3 × 3 square patches in the FoE model. Fig. 8 shows the radar image with reduced co-channel interference and bright speckles. In Fig. 8, bright speckles are suppressed significantly by the FoE model.

### 3.2 Sea clutter estimation based on radar image

**Fig. 9:**
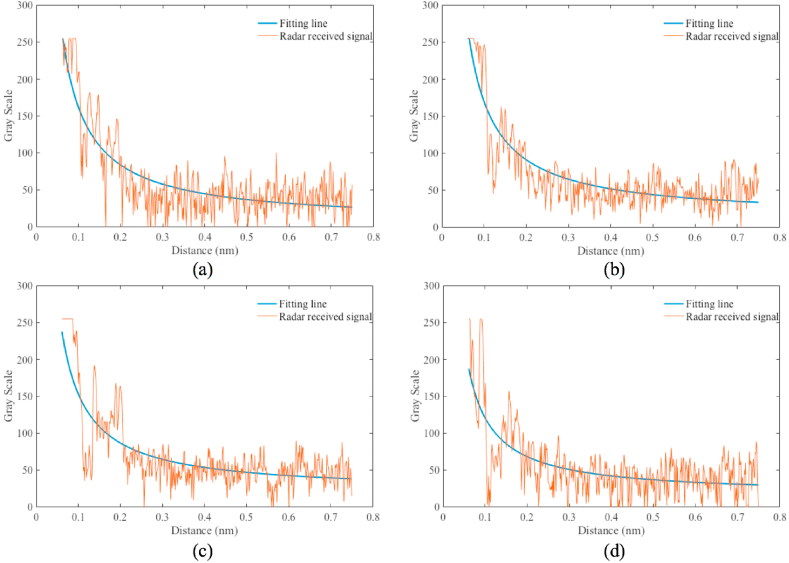
The fitting lines of the 4 incident angles, (a) the fitting line of ① in Fig. 8; (b) the fitting line of ② in Fig. 8; (c) the fitting line of ③ in Fig. 8; (d) the fitting line of ④ in Fig. 8.

**Fig. 10:**
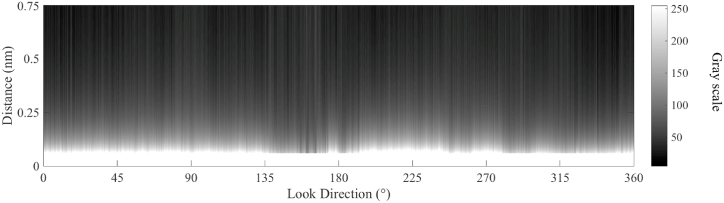
The fitting graph of the radar image according to the sea clutter fitting model.

**Fig. 11:**
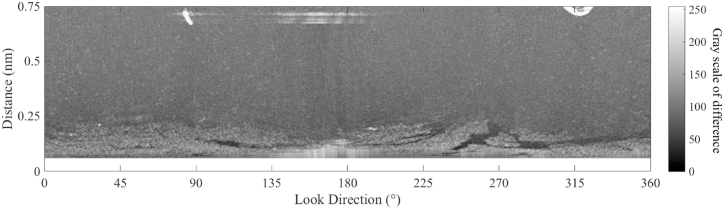
The difference of the fitting radar image and preprocessed radar image.

According to the sea clutter fitting model, in each look direction, a fitting line of the radar image in the Cartesian coordinate can be calculated. In the fitting calculation, M is set 3, and the radius of blind zone is 0.06 nautical mile. The fitting lines for the 4 look directions (the red line in Fig. 8) based on Equation (15) are shown in Fig. 9. As shown in Fig. 9, the fitting results are consistent with the power attenuation of the reflected signal from the sea clutter. In the positions of oil spills (from 0.11 nm to 0.12 nm and from 0.15 nm to 0.17 in Fig. 9a, from 0.10 nm to 0.13 nm in Fig. 9b, from 0.11 nm to 0.13 nm in Fig. 9c, from 0.10 nm to 0.11 nm in Fig. 9d), the value on the radar image is less than the fitting result. The fitted image of the sea clutter is shown in Fig. 10. The difference between the preprocessed radar image and the fitted radar image of sea clutter is used to detect the oil spills, and the value of the difference is scaled from 0 to 255, which is shown in Fig. 11.

### 3.3 Oil spill detection

**Fig. 12:**
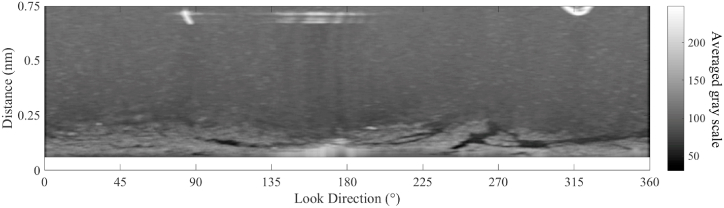
The processed radar image using a mean filter.

**Fig. 13:**
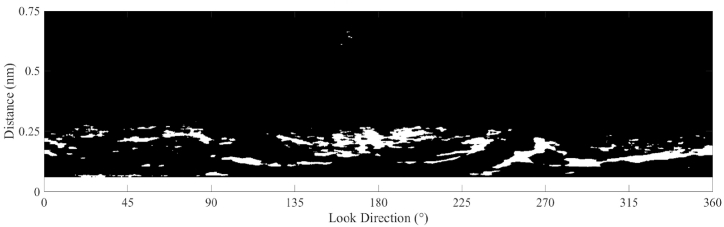
The oil spill detection by the Otsu method.

**Fig. 14:**
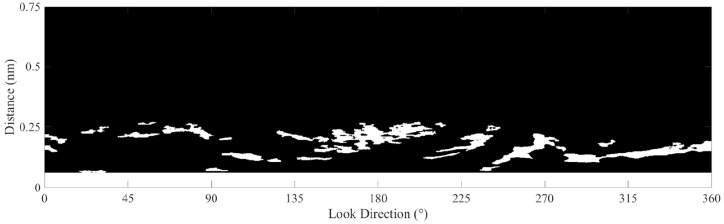
The denoised oil spill detection results.

**Fig. 15:**
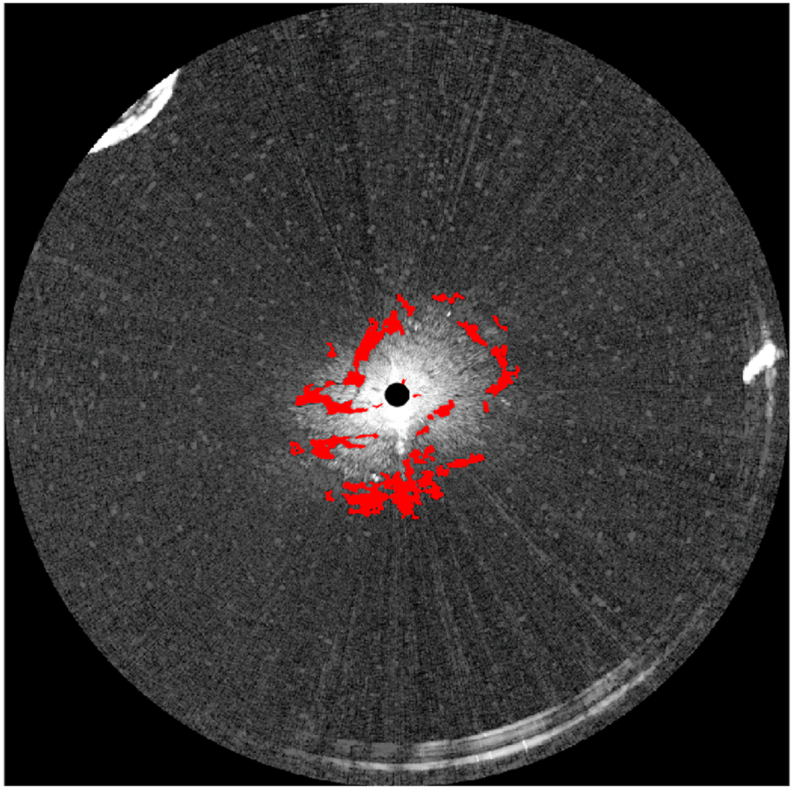
The detected oil spills (shown in red) marked on the marine radar image in the polar coordinate. (For interpretation of the references to color in this figure legend, the reader is referred to the Web version of this article.)

In marine radar images, oil spills decrease the intensity of the reflected signals [7], therefore, the dark areas in Fig. 11 are considered oil spill areas. The surface wave makes the reflected radar signal ruffled. A mean filter with a size of 5 × 20 is used, and the smoothened radar image is shown in Fig. 12. The Otsu method was used to obtain oil spill information based on the difference between the preprocessed radar image and the fitted radar image. The areas of oil spills detected by the Otsu method in the radar image are shown in Fig. 13. In Fig. 13, the white area in the radar image is the detected oil spill region. The oil spill area floating on the sea surface is considered a continuous zone. If the detected oil spill areas were small and not continuous, they were determined to be incorrect extractions and removed as noise. After removing the small areas (less than 200 pixels), the final oil spill detection results (white area) are shown in Fig. 14. As shown in Fig. 14, the sea clutter fitting model of a radar image could be used to detect the oil spills near the ship. The detected oil spills (in red color) plotted on the original sampled radar image is shown in Fig. 15.

## 4 Discussion

### 4.1 Oil spill event

**Fig. 16:**
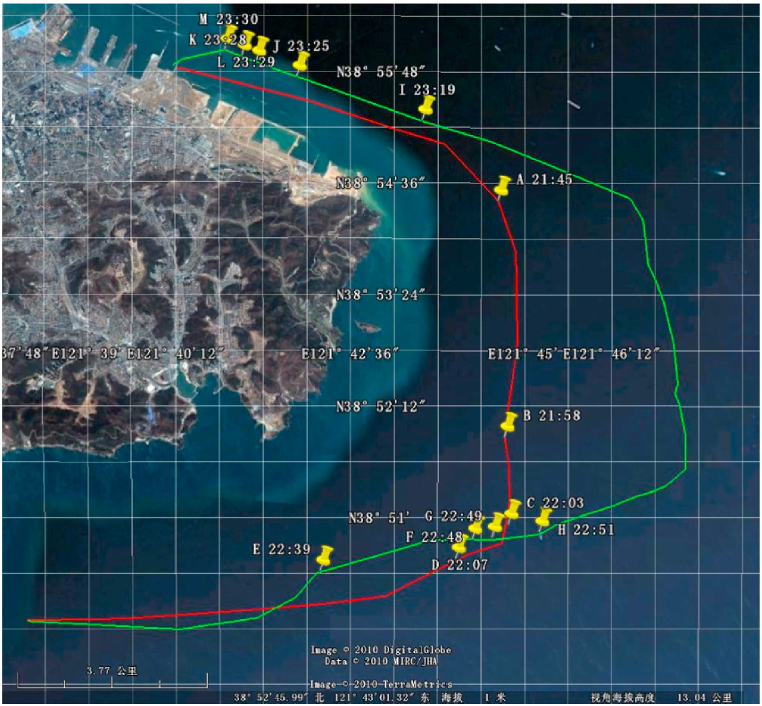
Navigation route of teaching-training ship “YUKUN” on July 21, 2010.

An oil spill accident happened on the Dalian coast, China, on July 16, 2010. Dalian Maritime University dispatched teaching-training ship “YUKUN” to carry out the oil spill detection in the coastal area. Radar images of an X-band marine radar provided by Sperry Marine were adopted to extract the oil spill information. Some parameters of the X-band marine radar are shown in Table 1. The navigation route for oil spill detection on July 21, 2010 is shown in Fig. 16. At 21:00 on July 21, 2010, the weather was cloudy, the wind speed was 5 km/h from the southwest, the temperature was 24 °C and the humidity was 92 %.

**Table 1:** Main parameters of X-band marine radar.

Name	Parameters
Working frequency	9.41 GHz
Antenna length	8 ft
Detection range	0.5–12 nautical miles
Horizontal direction	360 °
Vertical direction	± 10°
Peak power	25 kW
Pulse width	50 ns/250 ns/750 ns
Pulse repetition frequency	3000 Hz/1800 Hz/785 Hz
Blind zone	0.06 nautical mile

**Fig. 17:**
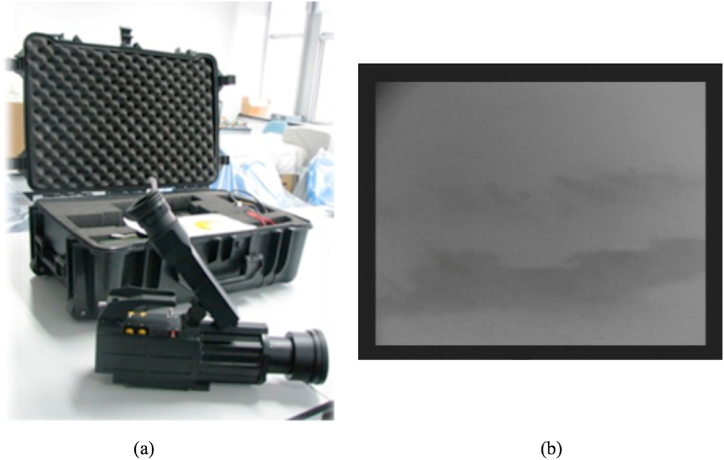
An oil spill image captured by thermal infrared sensor: (a) thermal infrared sensor; (b) captured thermal infrared image.

Oil spills and look-alikes (e.g., low-speed winds and internal waves) appear as dark patches in the radar images [33]. To exclude the look-alikes, a thermal infrared sensor (wavelength rang: 7.5–13 μm) was used. The thermal infrared sensor is shown in Fig. 17a, and a captured thermal infrared image is shown in Fig. 17b. The thermal infrared image in Fig. 7b is obtained at 23:19, 21 July 2010 on the teaching-training ship “YUKUN”. The temperature of the spilled oil at night is lower than the temperature of the water surface. Meanwhile, there will be no significant temperature difference in low-speed winds and internal wave areas. The thermal infrared image in Fig. 17b shows that the sea surface has low temperature areas (dark areas), indicating that the sampled radar images contain spilled oil instead of low-speed winds and internal waves.

For the sampled marine radar images, some other methods have been used to detect oil spills, such as the adaptive thresholding method, texture analysis, and machine learning. Sea clutter can also be estimated by polynomial fitting [34] and exponential fitting [35]. The machine learning method is fed by training data, which was affected by manual involvement. Therefore, oil spill detection by the adaptive thresholding method, polynomial fitting method and exponential fitting method were used to evaluate the performance of the proposed method in this paper.

### 4.2 Polynomial fitting

Regression is a numerical method to analyze the relationship between a dependent variable and one or more independent variables. Linear regression refers to a linear dependence on the unknown parameters to be estimated from the data.

The gray value of the n th pixel in each incident angle on the radar image can be expressed as:

(17) $$Gray\left(n\right)=\sum_{\eta =0}^{M}b_{\eta }n^{\eta }$$

where $b_{\eta }$ (η = 0, 1, 2, …, M) is the unknown parameter. For N pixels in the look direction, Equation (17) can be expressed as

(18) G=Aβ

where

$$G=\left[\begin{array}{c} Gray\left(1\right)\\ Gray\left(2\right)\\ \begin{array}{c} \vdots \\ Gray\left(N\right) \end{array} \end{array}\right]\;A=\left[\begin{array}{cc} \begin{array}{ccc} 1 & 1 & 1\\ 1 & 2 & 2^{2} \end{array} & \begin{array}{cc} \cdots & 1\\ \cdots & 2^{M} \end{array}\\ \begin{array}{ccc} \vdots & \vdots & \vdots \\ 1 & N & N^{2} \end{array} & \begin{array}{cc} \cdots & \vdots \\ \cdots & N^{M} \end{array} \end{array}\right]\;\beta =\left[\begin{array}{c} b_{0}\\ b_{1}\\ \begin{array}{c} \vdots \\ b_{M} \end{array} \end{array}\right]$$

One can determine the values of β in Equation (18) by means of the ordinary least square (OLS) method [34]. If the noise sampled by marine radar is Gaussian, then β can be obtained by

(19) $$\beta ={\left(A^{T}A\right)}^{-1}A^{T}G$$

### 4.3 Exponential fitting

An exponential fitting model [35] was used to estimate the power of marine radar receiver, which can be expressed as

(20) $$P_{r}=K_{s}R^{m}\text{,}$$

where $K_{s}$ and m are unknown quantities. To obtain the unknown quantities in Equation (20), ordinary least square method with Equation (19) is also used to calculate the parameters of $K_{s}$ and m in each incident angle.

### 4.4 Adaptive thresholding

The distance, roughness of the sea surface, and transmitting angle of the marine radar in horizontal affect the intensity of the reflected signal. Therefore, a one-fit-all threshold is inappropriate for oil spill extraction. An adaptive thresholding [[36], [37], [38], [39], [40]] widely used in image processing is adopted for oil spill detection. Bradley and Roth [41] proposed an effective adaptive thresholding method to extract desirable information in gray images.

In Bradley's method, the integral image in the first pass is calculated from the input image. In a second pass, the s×s average is computed using the integral image for each pixel in constant time, and then a comparison is drawn. If the value of the current pixel is t percent less than this average then it is set to black, otherwise, it is set to white.

### 4.5 Comparison and discussion

**Fig. 18:**
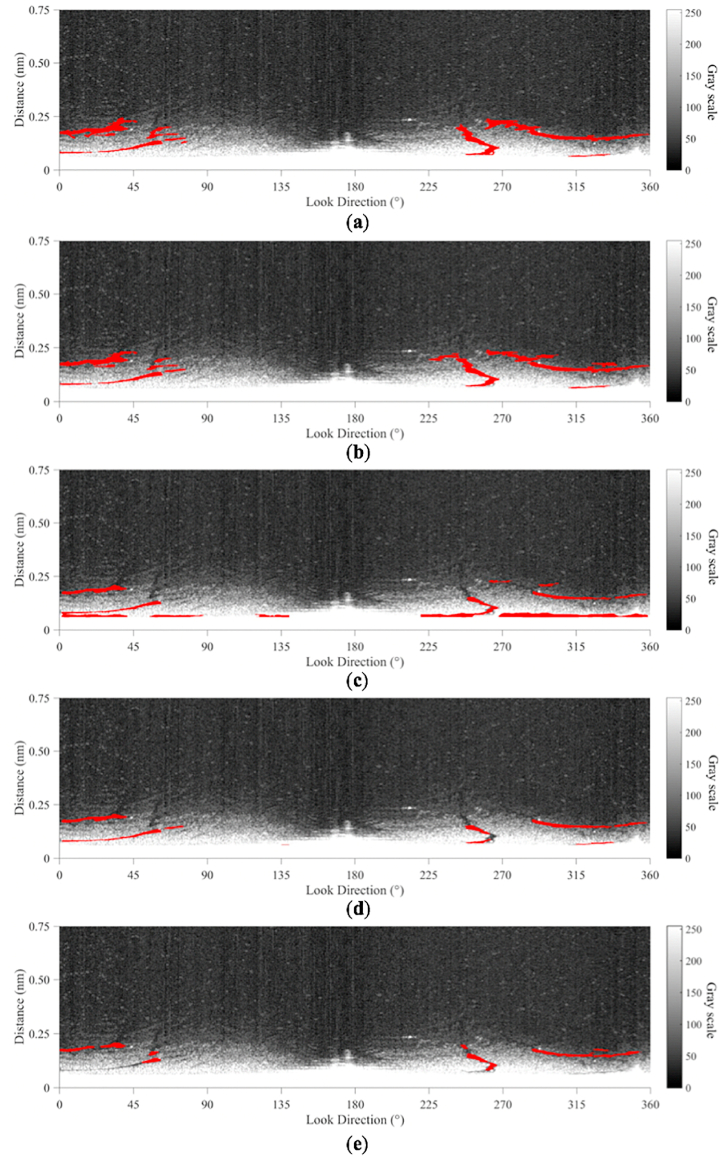
Comparison of oil spills segmentation on the radar image sampled at 23:19:24 by five methods: (a) visual interpretation; (b) sea clutter fitting method; (c) exponential fitting method; (d) polynomial fitting method; (e) adaptive thresholding method.

28 sampled radar images are used to carry out the comparison of five methods: visual interpretation, sea clutter fitting method, exponential fitting method, polynomial fitting method, and adaptive thresholding method. The oil detection results of the four methods on the radar image sampled at 23:19:24 on July 21, 2010 are shown in Fig. 18. Visual interpretation by experts is one of the most common methods for oil spill detection [42] and hence the visual interpretation is used as a reference value. In the sea clutter fitting method, the order of the negative exponent in the sea clutter fitting model is set at 5. The order of the polynomial fitting method is set at 10. In the adaptive thresholding method, the s is set to 32 and the t is set to 0.35 [17].

Fig. 18a is the visual interpretation results of oil spills. The oil spills extracted by the sea clutter fitting method in Fig. 18b are similar to the visual interpretation results. The exponential fitting method provides lots of misidentifications of oil spills at the bottom of the radar image in Fig. 18c. Some oil spills are missed by the polynomial fitting method and adaptive thresholding method in Fig. 18d and e.

**Fig. 19:**
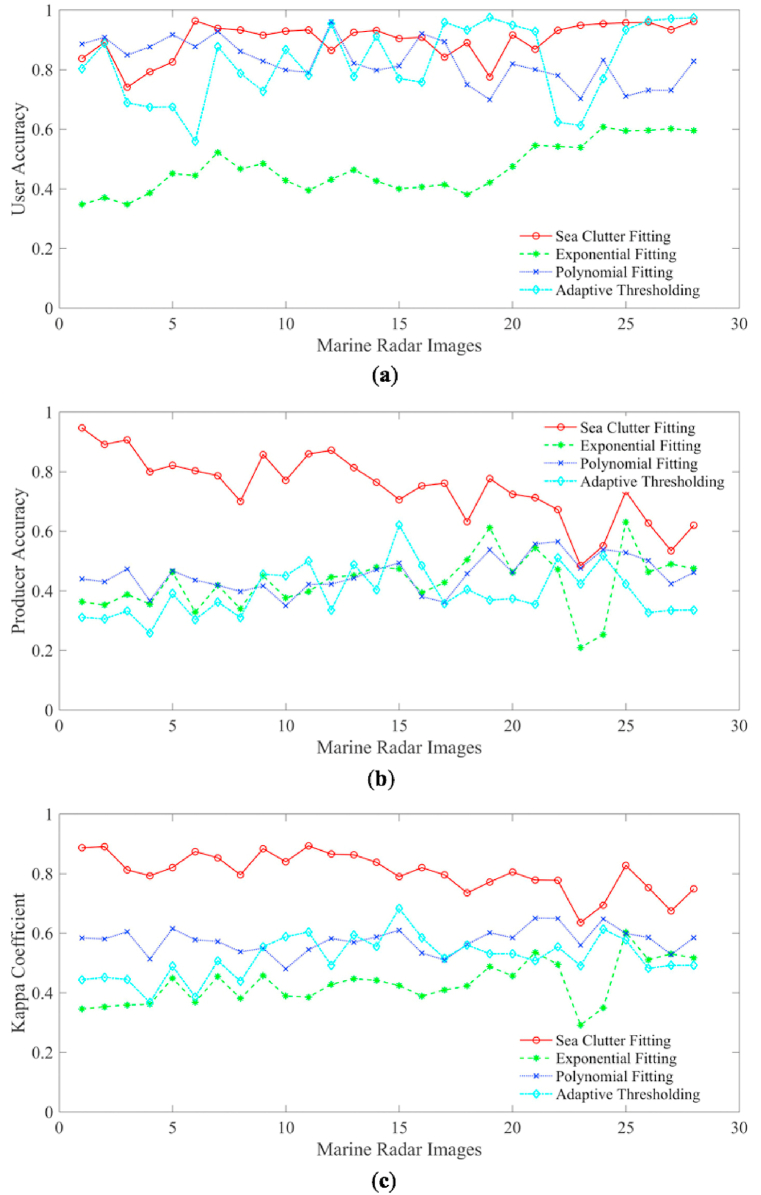
Accuracy of oil spill detection by the methods of sea clutter fitting, exponential fitting, polynomial fitting and adaptive thresholding. (a) User accuracy; (b) producer accuracy; (c) Kappa coefficient.

The user accuracy, producer accuracy, and Kappa coefficient of processed 28 radar images for oil spill detection by the four methods are shown in Fig. 19a, b and c respectively. The average results of user accuracy, producer accuracy, and Kappa coefficient by the four methods are shown in Table 2. The average user accuracy of the sea clutter method is 0.8993, which is a little larger than the polynomial fitting method and adaptive thresholding method, and much larger than the exponential method. The average producer accuracy of the sea clutter fitting method is 0.7459, which is much better than other three methods. The average Kappa coefficient of the sea clutter fitting method is 0.8044, which is the best one of the four methods.

**Table 2:** Average accuracy of 4 oil spill detection methods.

Methods	Average User Accuracy	Average Producer Accuracy	Average Kappa Coefficient
Sea Clutter Fitting	0.8993	0.7459	0.8044
Exponential Fitting	0.4675	0.4293	0.4304
Polynomial Fitting	0.8256	0.4538	0.5755
Adaptive Thresholding	0.8250	0.3946	0.5191

## 5 Conclusions

In this paper, we propose a sea clutter fitting model for oil spill detection. The sea clutter fitting model was derived based on the geometric structure of the marine radar, the expression of marine radar received power, and the rough surface scattering model. According to the sea clutter fitting model, the denoised radar image was processed to obtain the coarse distribution of oil spills. Finally, fine measurement of oil spills was carried out by the Otsu method and the restriction of oil spill areas.

The proposed method was used on the radar images sampled in an oil spill accident in Dalian on July 21, 2010. According to the proposed method, oil spills can be detected automatically. Compared with the methods using C–V levels, machine learning methods, texture analysis, and the adaptive thresholding method, the proposed method does not require human intervention. Compared with the artificial intelligence methods, the proposed method does not need additional training data, and the detected oil spills by the proposed method are not affected by the bias of training dataset. The oil spill detection accuracy of sea clutter fitting method is better than polynomial fitting method, exponential fitting method and adaptive thresholding method, and the oil spill detection results were consistent with visual interpretation.

In this research, oil spill extraction was carried out under conditions of known oil spills with the assistance of infrared detectors. When the oil spill information was unknown, additional analysis, such as geometrical, textural, and polarimetric features, should be added to determine the extracted dark patches as oil spills. Though the proposed model detects oil spills well in areas with high brightness, it fails to warn people about oil spills in remote areas. Future work remains to be done in this respect.

## Fundings

This research was funded by 10.13039/501100001809National Natural Science Foundation of China (52271359), by Fundamental Research Funds for the Central Universities (3132023126).

## Data availability statement

The data associated with our study have not been deposited in a publicly available repository. Data will be made available on request.

## CRediT authorship contribution statement

**Peng Liu:** Conceptualization, Data curation, Formal analysis, Funding acquisition, Investigation, Methodology, Validation, Visualization, Writing – original draft, Writing – review & editing. **Bingxin Liu:** Data curation, Formal analysis, Investigation, Methodology, Writing – original draft. **Ying Li:** Data curation, Resources. **Peng Chen:** Formal analysis, Investigation. **Jin Xu:** Formal analysis, Investigation.

## Declaration of competing interest

The authors declare that they have no known competing financial interests or personal relationships that could have appeared to influence the work reported in this paper.
